# Subwavelength Acoustic Valley-Hall Topological Insulators Using Soda Cans Honeycomb Lattices

**DOI:** 10.34133/2019/5385763

**Published:** 2019-08-08

**Authors:** Zhiwang Zhang, Ye Gu, Houyou Long, Ying Cheng, Xiaojun Liu, Johan Christensen

**Affiliations:** ^1^Department of Physics, MOE Key Laboratory of Modern Acoustics, Collaborative Innovation Center of Advanced Microstructures, Nanjing University, Nanjing 210093, China; ^2^Department of Physics, Universidad Carlos III de Madrid, 28916 Leganés, Madrid, Spain

## Abstract

Topological valley-contrasting physics has attracted great attention in exploring the use of the valley degree of freedom as a promising carrier of information. Recently, this concept has been extended to acoustic systems to obtain nonbackscattering sound propagations. However, previous demonstrations are limited by the cut-off frequency of 2D waveguides and lattice-scale size restrictions since the topological edge states originate from Bragg interference. Here we engineer topologically valley-projected edge states in the form of spoof surface acoustic waves that confine along the surface of a subwavelength honeycomb lattice composed of 330-mL soda cans. The inversion symmetry is broken through injecting a certain amount of water into one of the two cans in each unit cell, which gaps the Dirac cone and ultimately leads to the topological valley-Hall phase transition. Dual-frequency ranges of the valley-projected edge states below the sound line are observed, which originate from the first-order and second-order resonances, respectively. These results have the potential to enable promising routes to design integrated acoustic devices based on valley-contrasting physics.

Valleytronics [1–8], as a portmanteau of valley and electronics, was proposed to achieve the quantum manipulation over the valley degree of freedom, towards establishing a valley-spin-based technology that enables quantum computation [3, 4], valley selective excitation [5, 6], and topologically protected edge states [7, 8]. Over the past few years, this concept has also stimulated the analogous search for the topological valley-contrasting physics in classical systems, such as optics [9–12], acoustics [13–20], and mechanics [21–25]. Lu* et al.* introduced the vortex nature of the pseudospin valley states into the phononic crystal (PnC) [13] and experimentally demonstrated the existence of the topologically valley-projected edge states (TVPES) [14]. To extend the concept of topological order to the functional devices, a reconfigurable acoustic delay line [16] and a duplex directional acoustic antenna [17] based on TVPES have been experimentally demonstrated. However, all of these previous studies about TVPES entail several limiting factors. First, the aforementioned systems have been tested in closed waveguides of finite heights, which naturally sets a bound to the spectral window induced by the inevitable cut-off frequency. Second, sound waves can only be manipulated on lattice scales comparable to the operation wavelength as the TVPES originate from Bragg interferences. Consequently, systems based on subwavelength lattices supporting TVPES in open space remains an elusive and unanswered key question in the community. On the other hand, Lerosey* et al.* proposed a kind of soda cans metamaterial [26–28], which acts as Helmholtz resonators at deep-subwavelength scales, to obtain spoof surface acoustic waves (SAWs) [29, 30]. Topological edge states analogous to the quantum spin-Hall effect have been theoretically demonstrated [31] in such system composed of soda cans metamaterial. However, to our knowledge, an experimental verification of topological edge states confined above the soda cans has yet not been reported.

In this work, we design a honeycomb lattice [32, 33] composed of 330-mL soda cans arrangement in free space, which supports a dual-frequency Dirac cone at the corner of the first Brillouin zone (BZ) near both the first-order and second-order resonance at the same time. We find that these two Dirac cones are both located below the sound line, which guarantees the propagation of spoof SAWs. By breaking the inversion symmetry of the unit cell by injecting a certain amount of water into one of the two cans in each cell, the degeneracy of the valley states is lifted and two complete band gaps can be obtained. We demonstrate that the band inversions take place when introducing different symmetry breaking schemes, which can be characterized by the topological valley phase transitions. Furthermore, following the bulk-edge correspondence [34], we experimentally verify the existence of TVPES between two soda can arrays of opposite valley-Chern indices and illustrate its robustness against sharp bends.

Let us start with a discussion on the Helmholtz-like soda can resonator.  Figure 1(a) illustrates the basic schematic of such can having a height *H* = 13.02 cm, the radius *R* = 2.84 cm, and the neck length *h*_n_ = 0.2 cm. From the top view of the soda can, the neck can be considered as an ellipse with *d* = *r*_1_ = 1.25 cm and *r*_2_ = 0.75*r*_1_. The resonant frequency of the soda can is experimentally measured according to the standard test method ASTM E2611-09, of which the experimental setup is shown in Figure 1(b). As seen in Figure 1(c), the simulated first-order and second-order resonances are found near 402 Hz and 1465 Hz, respectively, which agree very well to corresponding experimental measurements: 402 Hz and 1442 Hz.

To achieve the single Dirac cone in the subwavelength scale, which is the necessary condition of the topological valley phase transition, the honeycomb PnC composed of multiple soda cans embedded in air is constructed as shown in Figure 2(a). Figure 2(b) shows the unit cell of PnC in the simulations containing two soda cans of heights *H*_A_ and *H*_B_. The center-to-center distance between the two cans is *D* = 6.6 cm and the lattice constant is $a=\sqrt{3}D$, which is 0.13*λ*_1_ and 0.49*λ*_2_ with *λ*_1_/*λ*_2_ the corresponding wavelength of the first/second resonance, respectively. The velocity and mass density of air are *c*_0_ = 343 m/s and *ρ*_0_ = 1.21 kg/m^3^. From the band diagrams in Figure 2(c), two single Dirac cones can be observed at the corners of the 1st BZ near the first-order and second-order resonance when the system is unperturbed (see gray circles in insets). Note that both Dirac cones are located below the sound line, which signifies the propagation of spoof SAWs confined to the array. However, in order to open the degeneracy, a perturbation should be introduced to break the inversion symmetry in the unit cell. Here the height difference between two cans is defined as Δ*H* = *H*_A_ − *H*_B_. In practical terms though, we inject water into the cans to change the effective height. Owing to the inexistence of the inversion symmetry after introducing Δ*H* = 0.2 cm, which means injecting 5-mL water into the can B only with A unchanged, the degenerated valley states based on the resonance of different orders are lifted to open a bulk band gap at the same time (see red dots in insets). As a result, two pairs of valley states (K_1_/K_2_ and K_3_/K_4_) exist at the K point below the sound line. Distributions of the total pressure fields and the sound intensity of four valley states are illustrated in Figure 2(d). The intensity vector is defined as **I** = (1/2)*p ***v**, where* p* represents the pressure amplitude and the vector **v** represents the sound velocity derived from the acoustic momentum equation. Three points should be noted. (i) The distributions of total pressure fields verify that the valley states K_1_/K_2_ are induced by the first-order resonance and K_3_/K_4_ are based on the second-order resonance. (ii) The sound intensity is strongly confined at the interface between the cans and free space, which verifies the propagation of spoof SAWs along the surface of the can array. (iii) The sound intensity of these valley states possesses the intrinsic circular polarized orbital angular momentum, which is also known as valley pseudospins. At K_1_ and K_3_ states, the sound field is left-handed circular polarized (LCP). Conversely, the acoustic vortex chiralities are right-handed circular polarized (RCP) at K_2_ and K_4_ states. Although just the eigenstates at K valley are considered here, we demonstrate that the counterparts at K′ valley possess invariant vortex but opposite chirality because of the time-reversal symmetry, which are not shown.

We demonstrate that the vortex chiralities of the corresponding valley states will be inverted if the inversion symmetry is introduced oppositely, for instance, changing the sign of Δ*H* to Δ*H* = −0.2 cm by injecting 5-mL water into the can A only with B unchanged. In Figure 3(a), eigenmodes of K_1_ and K_2_ valley states with different signs of Δ*H* are illustrated. The inversion of the chiralities can be clearly seen by means of the band inversion. Figure 3(b) shows that the eigenfrequencies of the valley states K_1_/K_2_ separate the band gap at K point as a function of Δ*H*. The purple and green lines with dots represent the valley states with LCP and RCP chiralities, respectively. Note that the chirality/band inversions can be also observed between K_3_ and K_4_ valley states, which are induced by the second-order resonance, as shown in Figures 3(c) and 3(d). As a result, the dual band inversions below the sound line are obtained in the proposed soda can PnC. Further, we demonstrate that the band inversion leads to a topological phase transition between different valleys, of which the topological invariant is described by valley-Chern indices. Derived from the** k**·**p** perturbation method, the effective Hamiltonian near the Dirac cone can be described as [14]

(1) $$\begin{array}{c} H=\nu_{D}\delta k_{x}\sigma_{x}+\nu_{D}\delta k_{y}\sigma_{y}+m\nu_{D}^{2}\sigma_{z}, \end{array}$$

where *ν*_D_ is the group velocity, *δ ****k*** = (*δk*_*x*_, *δk*_*y*_) is the distance from the Dirac points, *σ*_*i*_  (*i* = *x*, *y*, *z*) is Pauli matrices of the vortex, and *m* is the effective mass defined as *m* = (*ω*_RCP_ − *ω*_LCP_)/2*ν*_D_^2^. After the integral of the nontrivial Berry curvature over an individual valley, the nonvanishing valley-Chern indices can be determined by [8, 14]

(2) $$\begin{array}{c} C^{K}=\frac{1}{2}\times sgn\left(\;m\;\right). \end{array}$$

From (2) and Figure 3, we can conclude that the sign of the effective mass depends on the sign of height difference Δ*H*, which is shown in Figures 3(b) and 3(d). Furthermore, the valley indices of structure I (Δ*H* < 0) and structure II (Δ*H* > 0) are C_I_^K^ = −1/2 and C_II_^K^ = 1/2, respectively.

According to the bulk-boundary correspondence, helical edge states can exist at the interface where the effective mass changes sign. For example, the difference of the topological valley indices across the interface I-II is ΔC_I−II_^K^ = C_I_^K^ − C_II_^K^ = −1, which supports an edge state projected by the K valley propagating with negative group velocity. Correspondingly, the edge state projected by the K valley has a positive group velocity along the interface II-I with ΔC_II−I_^K^ = 1. To verify the above predictions, the band diagrams of a ribbon structure with zigzag-type interface composed of 6 unit cells with different topological valley phase at either side are illustrated in Figure 4. A corresponding dispersion relation of the ribbon-shaped PnC with the armchair-type interface is discussed in the Supplementary Material Note I. The first-order resonance induced edge states are shown in Figures 4(a)–4(c). For the interface I-II (blue dots), a backward-moving edge state projected by K valley has been predicted as shown in Figure 4(b). On the other hand, a forward-moving edge state projected by K valley is sustained along the interface II-I (red dots) as shown in Figure 4(c). Both results are good agreement with theoretical predictions. In addition, the edge states are not gapless in this situation, which is different from previous reports [14, 16]. We demonstrate that the soda can PnC with the perturbation |Δ*H*| = 0.2 cm is distorted to a relatively large level, which leads to the existence of the gapped valley-projected edge states. Note that the same perturbation has a stronger influence on the edge states pertaining to the second-order resonance whose band diagrams and the eigenmodes with different types of interfaces are illustrated in Figures 4(d)–4(f). Interestingly, the band of the edge state for the I-II seen in Figure 4(d) is almost entirely flat, which supports ultra-slow spoof SAW propagations useful for topological acoustic buffering [16].

To experimentally verify the existence of the TVPES, we construct a PnC composed of 12*a* × 12*a* soda can arrays as shown in Figure 5(a). The structures with opposite valley-Hall phases, which consist of red/green soda cans, are separated by a straight interface. In the simulations, one monopolar point source, which is marked by a red star in Figure 5(b), is placed at the left termination of the interface with a distance of 0.5 cm to the surface of the soda cans. The experimental details can be found in Supplementary Material Note II. Figure 5(b) illustrates the simulated absolute pressure fields at frequency *f* = 401.8 Hz within the topological band gap. The sound waves transport along the interface and decay exponentially into the bulk. To experimentally verify it, we measure the normalized sound energy along a cutline in the middle of the PnC [red dashed line in Figure 5(b)], which is illustrated in Figure 5(c) together with the simulated results. Note that the distance between the* y*-direction cutline and the soda can arrays is 0.5 cm and the measured cutline crosses over the mouths of 12 soda cans, of which the experimental details are illustrated in the Supplementary Material Note II. As can be observed, the energy peak at the interface and the good agreement between the simulated and experimental results confirm the existence of the TVPES. The in-plane energy decay lengths towards two regions, which can be observed from Figure 5(c), are 0.15*λ* and 0.11*λ*, indicating the tight confinement of TVPES at the interface. The slight disagreement between the predicted and observed energy profile results from the scattering by the experimental environment. Furthermore, we measure the amplitude of the acoustic pressure fields along the* z* direction away from one soda can, which is near the interface and indicated by a yellow ellipse in Figure 5(b). From the simulated and measured results at frequency* f* = 401.8 Hz shown in Figure 5(d), we demonstrate that the acoustic field is indeed confined to the surface and decays into free space exponentially, verifying the surface confined nature of the spoof SAW propagating in the form of a TVPES.

The hallmark of the physical properties of topological edge states is its robustness against defects as we will discuss in the following.  Figure 6(a) illustrates the PnC with a 120°-bended interface between two regions. The simulated distributions of the pressure fields at frequency* f* = 401.8 Hz indicate that sound waves transport along the route without backscattering even when the sharp bend is introduced. In the experiment, we measure the absolute pressure profiles along a cutline as shown in Figure 6(a), of which the data are illustrated in Figure 6(b) together with the simulated results. The agreement between the simulated and the experimental results verifies the robustness of the TVPES against the sharp bend. We demonstrate that the slightly increased pressure amplitude near the top termination of the cutline results from the scattering induced by the experimental environment. To further corroborate the robustness of the system, we introduce two 120° sharp bends into a larger PnC composed of a 16*a* × 16*a* soda cans array.  Figure 6(c) shows the simulated absolute pressure fields, from which sound waves transporting along the bended route without backscattering can be clearly observed. TVPES induced by the second-order resonance is discussed in Supplementary Material Note III.

To conclude, we have demonstrated a spoof SAW-based subwavelength valley-Hall topological insulator by arranging a honeycomb pattern of soda cans in open space. The topological valley-Hall phase transitions can be generated when the inversion symmetry is broken, simply by injecting water into the cans. Our experimental verification unequivocally demonstrates how spoof SAWs in soda cans lattices can confine along the interface between two distinct insulators in the form of topologically valley-projected edge states. Furthermore, the robustness against two sharp bends reassures the resilience of these novel surface waves. The proposed results may provide yet unseen possibilities to employ topological valley-contrasting acoustics in free space for audible sound control and guiding.

## Figures and Tables

**Figure 1:**
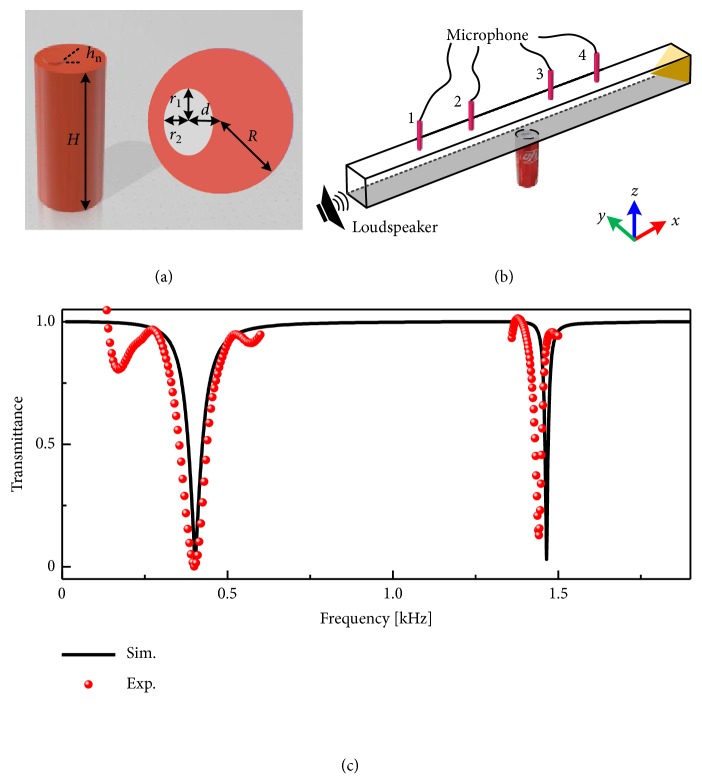
(a) Schematic of the soda can in the simulations. Right panel: top view of the structure. (b) Measurement system of sound transmittance after inserting a soda can in the waveguide. Sound waves are excited at one side of the waveguide and are absorbed by acoustic sponge at the other side. (c) Simulated and experimental measured transmittance.

**Figure 2:**
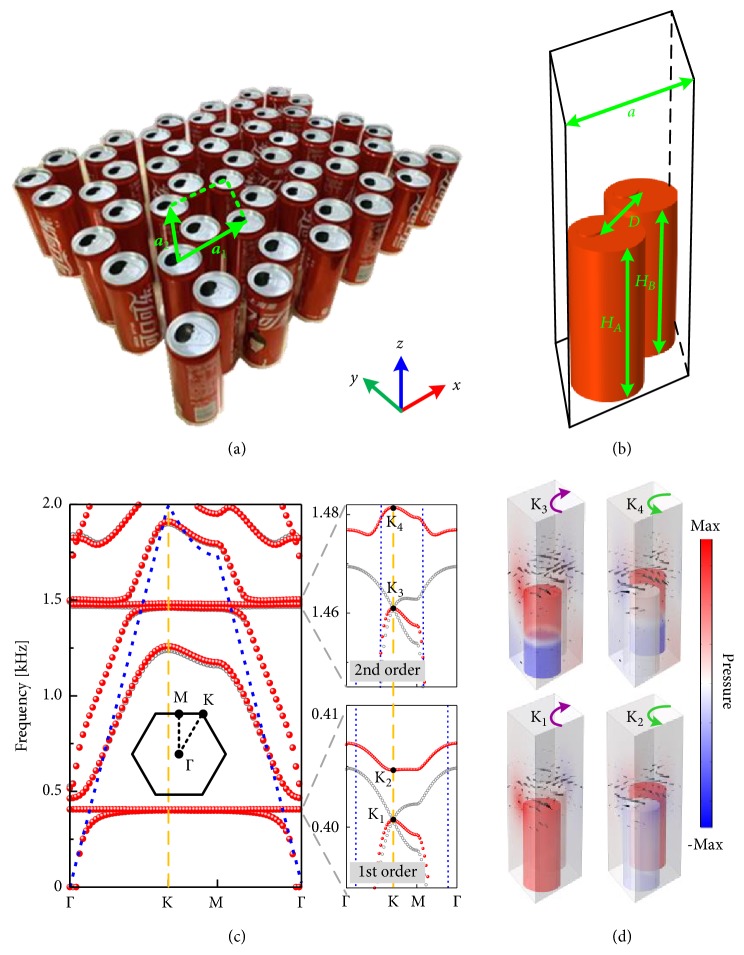
(a) Schematic of the honeycomb lattice consisting of soda cans with basis vectors **a**_1_ and **a**_2_. (b) Schematic of the unit cell in the simulations. (c) Dispersion relations of PnC without/with perturbation. Right panel: zoom in band diagram near the frequency of the first-order resonance and the second-order resonance. K_1_, K_2_, K_3_, and K_4_ denote four valley states at K point correspondingly. Gray circles and red dots represent the situations with Δ*H* = 0 and Δ*H* = 0.2 cm, respectively. Blue dashed line represents the sound line. (d) Eigenmodes of four valley states. Colors represent total pressure fields and the black cones show the direction and amplitude of the intensity.

**Figure 3:**
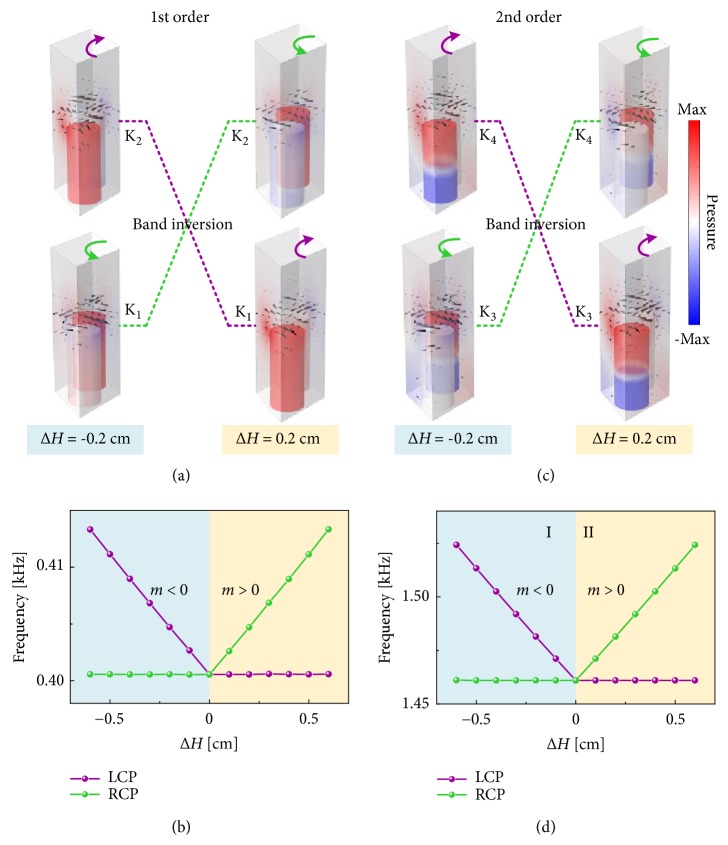
(a) Band inversions between K_1_ and K_2_ states induced by the 1st-order resonance with changing the perturbation from Δ*H* = −0.2 cm to Δ*H* = 0.2 cm. (b) Eigenfrequency evolution of the valley states K_1_/K_2_ depending on Δ*H* at the K point. (c)-(d) Same as (a)-(b) but of the valley states K_3_ and K_4_ induced by the 2nd-order resonance. The cyan and yellow regions show two different valley-Hall phases, labeled as I and II.

**Figure 4:**
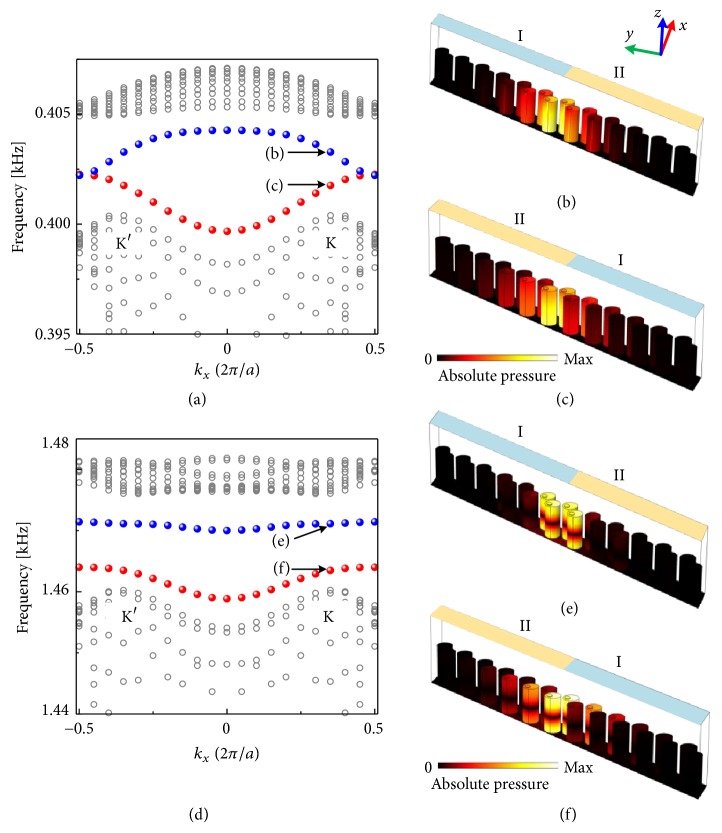
(a) Dispersion relation of the ribbon-shaped PnC near the frequency of the first-order resonance with different interfaces (I-II and II-I) comprised of 6 cells on either side. Gray circles represent bulk states and red/blue dots represent the edge states along different interfaces. Distributions of the absolute pressure fields at *k*_*x*_ = 0.35 × 2*π*/*a* for the interface (b) I-II and (c) II-I, respectively, which are labeled in (a). (d)-(f) Same as (a)-(c), but induced by the second-order resonance.

**Figure 5:**
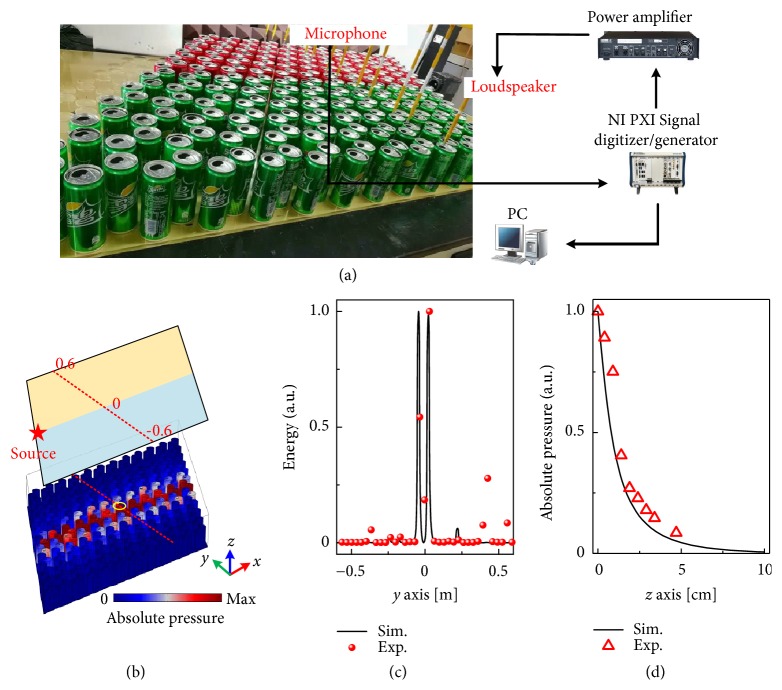
(a) Photograph of the experimental setup. (b) Simulated distributions of the absolute pressure fields along the straight interface with the frequency *f* = 401.8Hz within the band gap. (c) Experimental measured sound energy profile and the simulated results along the cutline in the middle of the crystal [red dashed line in (b)], which are labeled as red dots and black curve, respectively. (d) Relationship between the acoustic pressure amplitude above the soda can, which is chosen as the one near the interface labeled as yellow ellipse in (b), and the distance away from the surface of the soda can arrays in the vertical direction.

**Figure 6:**
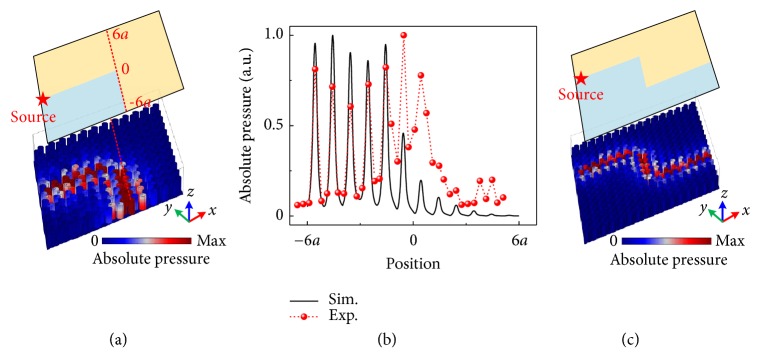
(a) Simulated distributions of the absolute pressure fields along the 120°-bended interface with the frequency* f* = 401.8 Hz. (b) Simulated and experimental measured pressure amplitudes along the cutline labeled by red dashed line in (a). Black solid curve and red dotted curve represent the simulated and the experimental results, respectively. (c) Simulated distributions of the absolute pressure fields along the interface with two 120° bends in a larger PnC.

## Supplementary Materials

Supplementary Materials
Figure 1: (a) dispersion relation of the ribbon-shaped PnC with the armchair-type interface near the frequency of the first-order resonance. Gray circles represent bulk states and red dots represent the edge states; (b)-(c) distributions of the absolute pressure fields at *k*_*x*_ = 0.05 × 2*π*/*a*_0_. Figure 2: photographs of the placements of (a) the loudspeaker and (b) the microphones in the experiments. Figure 3: simulated distributions of the absolute pressure fields at the frequency *f* = 1463.5 Hz (a) along the straight interface and (b) along the bended interface, respectively.
