# Supplementary material for: Subwavelength Acoustic Valley-Hall Topological Insulators Using Soda Cans Honeycomb Lattices
Source: Research (Wash D C). 2019 Aug 8;2019:5385763. doi: 10.34133/2019/5385763 (PMC6750043; doi:10.34133/2019/5385763)
Supplement: Supplementary Materials — Figure 1: (a) dispersion relation of the ribbon-shaped PnC with the armchair-type interface near the frequency of the first-order resonance. Gray circles represent bulk states and red dots represent the edge states; (b)-(c) distributions of the absolute pressure fields at kx = 0.05 × 2π/a0. Figure 2: photographs of the placements of (a) the loudspeaker and (b) the microphones in the experiments. Figure 3: simulated distributions of the absolute pressure fields at the frequency f = 1463.5 Hz (a) along the straight interface and (b) along the bended interface, respectively. [file 5385763.f1.pdf]

Supplementary Material for:
Subwavelength acoustic valley-Hall topological insulators using soda cans honeycomb lattices

Zhiwang Zhang,[1,2] Ye Gu,[1] Houyou Long,[1] Ying Cheng,[1,a)] Xiaojun Liu,[1,b)] and Johan Christensen[2,c)]

[1)] *Department of Physics, MOE Key Laboratory of Modern Acoustics, Collaborative Innovation Center of Advanced Microstructures, Nanjing University, Nanjing 210093, China*

[2)] *Department of Physics, Universidad Carlos III de Madrid, ES-28916 Leganés, Madrid, Spain*

(Dated: 28 June 2019)

[a)] Electronic mail: chengying@nju.edu.cn

[b)] Electronic mail: liuxiaojun@nju.edu.cn

[c)] Electronic mail: johan.christensen@uc3m.es

I. DISPERSION RELATION OF THE RIBBON PNC WITH ARMCHAIR-TYPE INTERFACE

Figure 1(a) illustrates the band diagram of the ribbon structure with an armchair-type interface. The ribbon structure is composed of structure II on the top of structure I. The corresponding distributions of the absolute pressure fields at $k_x = 0.05 \times 2\pi/a_0$ are shown in Figs. 1(b) and 1(c), respectively. Two points should be noted: (i) The edge states confined at the armchair-type interface can be clearly observed in the band gap. It has also been proved that the edge state exists for arbitrary interface angle in elastic valley-Hall topological insulators[1]. (ii) The edge states propagating along armchair-type interface are projected by K and K' valleys at the same time, due to the 30°-rotation interface compared with the zigzag-type interface. Consequently, there exist two edge states in the band gap.

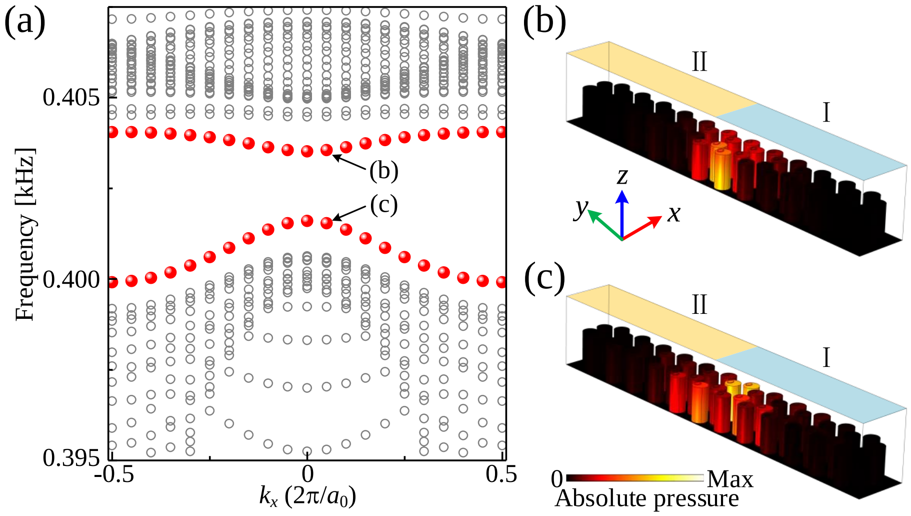

FIG. 1. (a) Dispersion relation of the ribbon-shaped PnC with the armchair-type interface near the frequency of the first-order resonance. Gray circles represent bulk states and red dots represent the edge states. (b)-(c) Distributions of the absolute pressure fields at $k_x = 0.05 \times 2\pi/a_0$.

II. EXPERIMENTS

The soda cans array in Fig. 5(a) consists of 288 soda cans embedded in an air matrix. In general, spoof SAWs are characterized by large parallel momentum to constitute strongly confined surface waves having an imaginary out-of-plane momentum. In order to excite these near-field waves a point source has to be placed in the nearest vicinity to the soda cans crystal. In doing this, the evanescent field containing large parallel momentum emanating the source is able to momentum match the near-field of the crystal. Accordingly, the microphones need also to be placed into the near-field of the crystal to be able to detect the spoof SAWs. To do so, experiments are conducted by one loudspeaker (PLS-75F24AL05-04) positioned very close to one of the terminations of the interface as shown in Fig 2(a). We emphasize that the loudspeaker can be analogous to a point source with the diameter of the loudspeaker $D = 6$ cm, which is 0.07 times compared with the corresponding wavelength. When measuring the sound energy amplitude in Figs. 5 and 6 of main text, the condensed microphones (GRAS type 40PH) are hung above the soda cans array from a distance to the surface of $d = 0.5$ cm as shown in Fig. 2(b). The outputs of the microphones are acquired by a digitizer (NI PXI-4498), and processed by LabVIEW software.

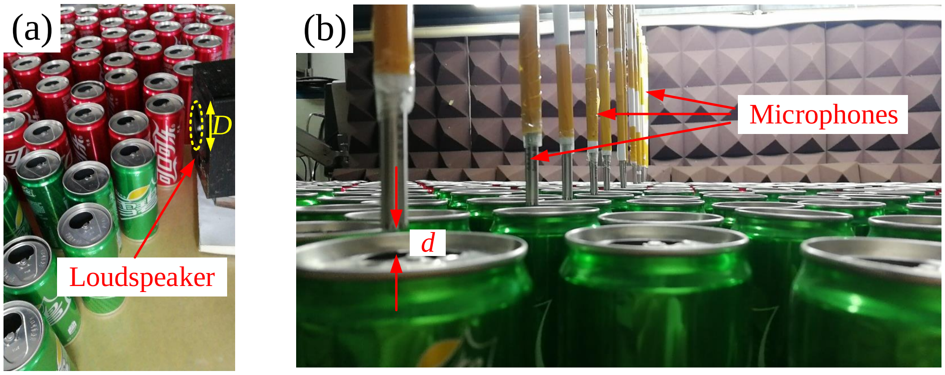

FIG. 2. Photographs of the placements of (a) the loudspeaker and (b) the microphones in the experiments.

III. TVPES INDUCED BY THE SECOND-ORDER RESONANCE

We demonstrate that the propagation of the second-order edge states which support the ultra-slow spoof SAW is confirmed as shown in Fig. 3. Figure 3(a) illustrates the simulated absolute pressure fields at the frequency $f = 1463.5$ Hz within the topological band gap near the second-order resonance. The sound waves excited by a point source at the left termination transport along the interface and decay exponentially into the bulk. Furthermore, the propagation along the bended path shown in Fig. 3(b) indicates good robustness against the sharp bend.

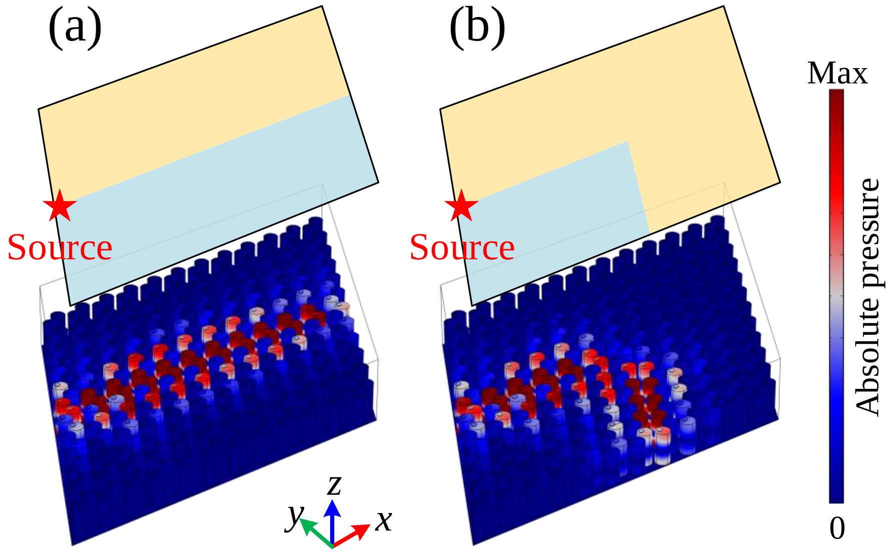

FIG. 3. Simulated distributions of the absolute pressure fields at the frequency $f = 1463.5$ Hz (a) along the straight interface and (b) along the bended interface, respectively.

REFERENCES

[1]Y. Chen, X. Liu, and G. Hu, J. Mech. Phys. Solids **122**, 54 (2019).
